# Mitochondrial DNA copy number is regulated by DNA methylation and demethylation of *POLGA* in stem and cancer cells and their differentiated progeny

**DOI:** 10.1038/cddis.2015.34

**Published:** 2015-02-26

**Authors:** W Lee, J Johnson, D J Gough, J Donoghue, G L M Cagnone, V Vaghjiani, K A Brown, T G Johns, J C St. John

**Affiliations:** 1 Centre for Genetic Diseases, MIMR-PHI Institute of Medical Research, Monash University, 27-31 Wright Street, Clayton, Victoria 3168, Australia; 2 Department of Molecular and Translational Science, Monash University, 27-31 Wright Street, Clayton, Victoria 3168, Australia; 3 Centre for Cancer Research, MIMR-PHI Institute of Medical Research, Monash University, 27-31 Wright Street, Clayton, Victoria 3168, Australia

## Abstract

Mitochondrial DNA (mtDNA) copy number is strictly regulated during differentiation so that cells with a high requirement for ATP generated through oxidative phosphorylation have high mtDNA copy number, whereas those with a low requirement have few copies. Using immunoprecipitation of DNA methylation on 5-methylcytosine (5mC) and 5-hydroxymethylcytosine (5hmC), which distinguish between *de nov*o DNA methylation and demethylation, respectively, we set out to determine whether DNA methylation at exon 2 of the human mtDNA-specific polymerase (DNA polymerase gamma A (*POLGA*)) regulates cell-specific mtDNA copy number in highly proliferative and terminally differentiated cells. Highly proliferative cancer and pluripotent and multipotent cells possessed low mtDNA copy number and were highly methylated at exon 2 of *POLGA* in contrast to post-mitotic cells. Unlike neural stem cells, cancer cells were unable to differentiate and remained extensively DNA methylated at exon 2 of POLGA. However, mtDNA depletion of cancer cells reduced DNA methylation at exon 2 of *POLGA* as they replenished mtDNA to form tumours in mice. Glioblastoma cells treated with the DNA demethylation agent 5-azacytidine over 28 days of astrocyte-induced differentiation demethylated exon 2 of *POLGA* leading to increased mtDNA copy number and expression of the astrocyte endpoint marker glial fibrillary acidic protein (*GFAP*). However, the demethylation agent vitamin C (VitC) was unable to sustain increased mtDNA copy number and differentiation, as was the case when VitC was withdrawn after short-term treatment. These data demonstrate that DNA demethylation of *POLGA* is an essential regulator of mtDNA copy number and cellular fate and that cancer cells are only able to modulate DNA methylation of *POLGA* and mtDNA copy number in the presence of a DNA demethylation agent that inhibits *de novo* methyltransferase 1 activity.

The human mitochondrial genome (mitochondrial DNA, mtDNA) is 16.6 kb, circular and double stranded. It encodes 13 polypeptides of the electron transfer chain, which generates the majority of cellular ATP through oxidative phosphorylation (OXPHOS). Of these genes, 12 are located on the heavy strand and one on the light strand. MtDNA also encodes 22 tRNAs and 2 rRNAs. The non-coding regions are the D-loop and regions interspersed between the coding gene and tRNAs on the light strand.^1, 2^ The D-loop is also the site of interaction for the nuclear-encoded mtDNA transcription and replication factors.

MtDNA copy number is cell type specific and dependent on the strict regulation of mtDNA replication during development.^3, 4^ Copy number increases progressively during oogenesis and reaches maximal levels in mature fertilisable oocytes.^4, 5^ Copy number is then significantly reduced during preimplantation development before gastrulation.^3^ These early cells remain undifferentiated and pluripotent with the potential to differentiate into all cell types of the body. Reduction in mtDNA copy number establishes the mtDNA set point, which then enables undifferentiated cells to accumulate sufficient numbers of mtDNA to facilitate their cell-specific requirements for OXPHOS-derived ATP.^6, 7^ Consequently, muscle cells and cardiomyocytes have high numbers of mtDNA copy, whereas endothelial and spleen cells have very few copies.^8, 9^

MtDNA replication is initiated by the nuclear-encoded mitochondrial transcription factor A. A by-product of this reaction is an RNA–DNA hybrid primer that is utilised by the nuclear-encoded DNA polymerase gamma A (POLGA), the catalytic subunit of POLG, which has polymerisation and exonuclease activities, to replicate mtDNA.^10, 11^ POLGA is assisted by POLGB, the accessory subunit, which stabilises the catalytic subunit to enhance fidelity. *PolgA* is the target of DNA methylation in mouse cells and tissues.^9, 12^

During mouse spermatogenesis, *PolgA* is DNA methylated at exon 2.^12^ Furthermore, in non-transformed mouse cells, DNA methylation negatively correlates with mtDNA copy number in a tissue-specific manner.^9^ This indicates that mtDNA copy number is regulated by DNA methylation of a mammalian nuclear-encoded gene and not of the mitochondrial genome. However, mouse induced pluripotent stem cells, derived from somatic cells, do not regulate *PolgA* in a similar manner. When induced to differentiate, they fail to increase mtDNA copy number and complete differentiation.^13^

Human embryonic stem cells (hESCs) are extensively DNA methylated, which is reduced during differentiation.^14, 15, 16^ They also progressively increase mtDNA copy number in a cell-specific manner, as do human neural stem cells (hNSCs).^17^ For example, at the completion of astrocyte differentiation, hNSCs possess significantly more copies of mtDNA, downregulate expression of multipotent neural genes, such as *NESTIN, MUSASHI1* and *CD133*, and upregulate expression of the endpoint marker glial fibrillary acidic protein (*GFAP*). However, cancer cells, such as those giving rise to Glioblastoma multiforme, the most aggressive malignant primary brain tumour,^18, 19^ are extensively DNA methylated.^20^ When induced to differentiate, they fail to increase mtDNA copy number and continue to express *NESTIN, MUSASHI1* and *CD133*.^17^ The maintenance of low mtDNA copy number promotes aerobic glycolysis for the generation of ATP, a combination of glycolysis with a small contribution from OXPHOS.^21, 22^ This promotes cell proliferation and self-renewal and prevents differentiation.^23, 24^

DNA demethylation agents, such as 5-azacytidine (5-azaC), have been used as therapeutic agents for cancer patients.^25, 26^ By demethylating the tumour genome, these agents promote cell differentiation to deplete the cancer cell pool.^27, 28, 29^ However, withdrawal results in cells returning to their semi-undifferentiated state, also known as the ‘rebound' effect.^30, 31^ Nevertheless, treatment of induced pluripotent stem cells with 5-azaC induces differentiation and synchronised increases in mtDNA copy number similar to ESCs.^13^

DNA methylation can be quantitatively assessed as *de novo* DNA methylation and demethylation by determining levels of enrichment for 5-methylcytosine (5mC) and 5-hydroxymethylcytosine (5hmC), respectively ^32^ through immunoprecipitation of methylated DNA (MeDIP).^33^ We have determined whether cancer cells are more extensively DNA methylated at exon 2 of *POLGA*, which would restrict mtDNA copy number to low levels and promote cell proliferation. To undertake this, we have used cancer cells, pluripotent and multipotent cells, and terminally differentiated cells and tissues. We show significantly greater enrichment for 5mC within *POLGA* in cancer cells, which prevents the synchronous increase in mtDNA copy number and results in stalled differentiation. However, the application of the global DNA demethylation agent, 5-azaC, demethylates cancer cells promoting mtDNA replication and differentiation.

## Results

### High levels of DNA methylation at exon 2 of *POLGA* correlate with low mtDNA copy number

To demonstrate that DNA methylation of human *POLGA* modulates mtDNA copy number, we performed bisulphite sequencing on 14 CpG dinucleotides within exon 2 of *POLGA* (NCBI: NM_001126131; chromosome 15, region 89876381–89876589). This region is homologous to the region in mouse *PolgA* that is DNA methylated.^9^ We analysed pluripotent (Mel-1), multipotent (hNSC) and cancer (HSR-GBM1 and U266) cell lines. Each of the cell lines was highly methylated (>90%) with no significant differences among the lines (Supplementary Figure S1).

**Figure 1:**
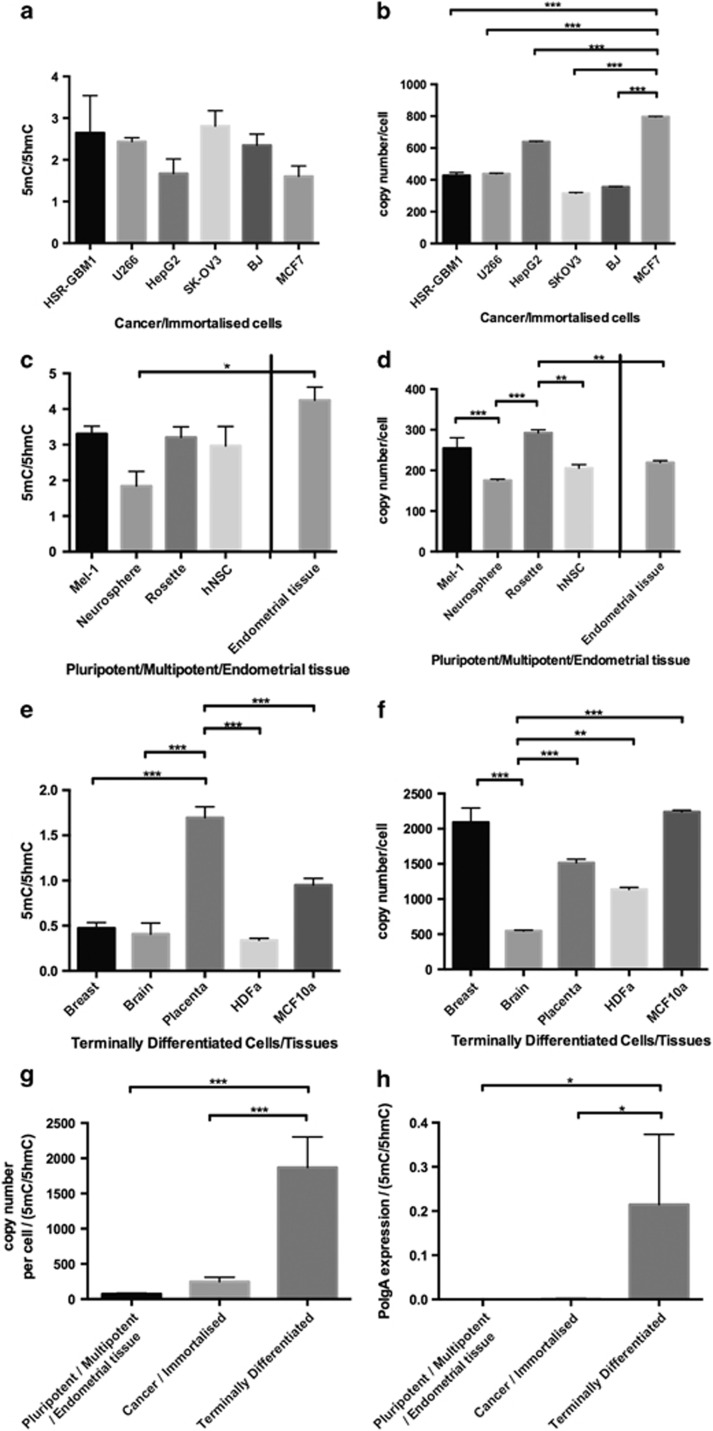
The ratios of enrichment of 5mC and 5hmC at exon 2 of *POLGA* and mtDNA copy number. (**a**) 5mC/5hmC ratios in cancer and transformed cells. (**b**) mtDNA copy number in cancer and transformed cells. (**c**) 5mC/5hmC in pluripotent/multipotent cells and endometrial tissue. (**d**) mtDNA copy number in pluripotent/multipotent cells and endometrial tissue. (**e**) 5mC/5hmC in terminally differentiated/post-mitotic tissues/cells. (**f**) mtDNA copy number in terminally differentiated/post-mitotic tissues/cells. (**g**) mtDNA copy number over ratios of 5mC/5hmC for all three categories of cells. (**h**) *POLGA* expression over ratios of 5mC/5hmC for all three categories of cells. Significance: *P<0.05; **P<0.01; ***P<0.001

As bisulphite sequencing does not distinguish between *de novo* DNA methylation and DNA demethylation,^34^ we performed MeDIP using antibodies to 5mC and 5hmC to determine their respective levels of enrichment. There were no significant differences among the cancer cell lines, HSR-GBM1 (Glioblastoma multiforme), U266 (multiple myeloma), HepG2 (hepatocarcinoma), SK-OV3 (ovarian cancer) and MCF7 (breast cancer), for levels of either 5mC or 5hmC. The mean ratio for 5mC:5hmC was 2.3±0.8 (Figure 1a), whereby enrichment for 5mC was twofold higher than 5hmC. We observed a similar outcome for the transformed fibroblast line, human skin fibroblast (BJ) (Figure 1a). Furthermore, mtDNA copy number was highest in MCF7 cells and lowest in SK-OV3 cells (*P*<0.001; Figure 1b), which is lower than for mature terminally differentiated cells.^35^

To determine if enrichment for 5mC and 5hmC and mtDNA copy number were similar for pluripotent and multipotent cells, we analysed the hESC line Mel-1, its neurosphere and rosette derivatives, and an hNSC line. These cell types can differentiate into mature cells with high mtDNA copy number. There were no significant differences between the ratios of 5mC and 5hmC for each of the cell types except the neurospheres (*P*<0.05; Figure 1c). Each of these cell types had fewer than 300 copies of mtDNA with the neurospheres (*P*<0.001) and hNSCs (*P*<0.01) possessing significantly fewer (Figure 1d). We also analysed endometrial tissues with this cohort, which are highly proliferative and were harvested at the most proliferative stage of the menstrual cycle. The profiles of enrichment for 5mC and 5hmC and mtDNA copy number were within range identified for pluripotent and multipotent cells (Figures 1c and d).

To further investigate the relationship between mtDNA copy number and DNA methylation at exon 2 of *POLGA*, we analysed terminally differentiated tissues and cells. These included breast, brain, placenta and the primary human fibroblast line, HDFa. Breast and brain tissues and fibroblast cells had significantly less enrichment for 5mC than the placental tissue (*P*<0.001; Figure 1e), which had a 5mC:5hmC ratio of 1.5. Nevertheless, breast tissue and breast epithelial cells (MCF10a) had significantly higher mtDNA copy number than brain tissue and fibroblast cells, which highlights the differences between terminally differentiated cell types.

Our outcomes are further highlighted by expressing mtDNA copy number as a function of 5mC:5hmC. The ratios for the pluripotent/multipotent and endometrial tissue, and cancer/immortalised cell groups were significantly lower than for the terminally differentiated group (*P*<0.01; Figure 1g). Consequently, exon 2 of *POLGA* is highly methylated within highly proliferative cancer, and pluripotent and multipotent cells and endometrial tissue, which correlates with low mtDNA copy number. In contrast, terminally differentiated cells and tissues, which proliferate at a much slower rate, have higher mtDNA copy number, whereas exon 2 of *POLGA* is methylated at lower levels. When we assessed the expression of *POLGA* as a function of 5mC and 5hmC at exon 2, the ratios for the pluripotent/multipotent and endometrial tissue, and cancer/immortalised cell groups were significantly lower than for the terminally differentiated group (*P*<0.05; Figure 1h).

### Changes in DNA methylation at exon 2 of *POLGA* in tumours derived from mtDNA-depleted cells

**Figure 2:**
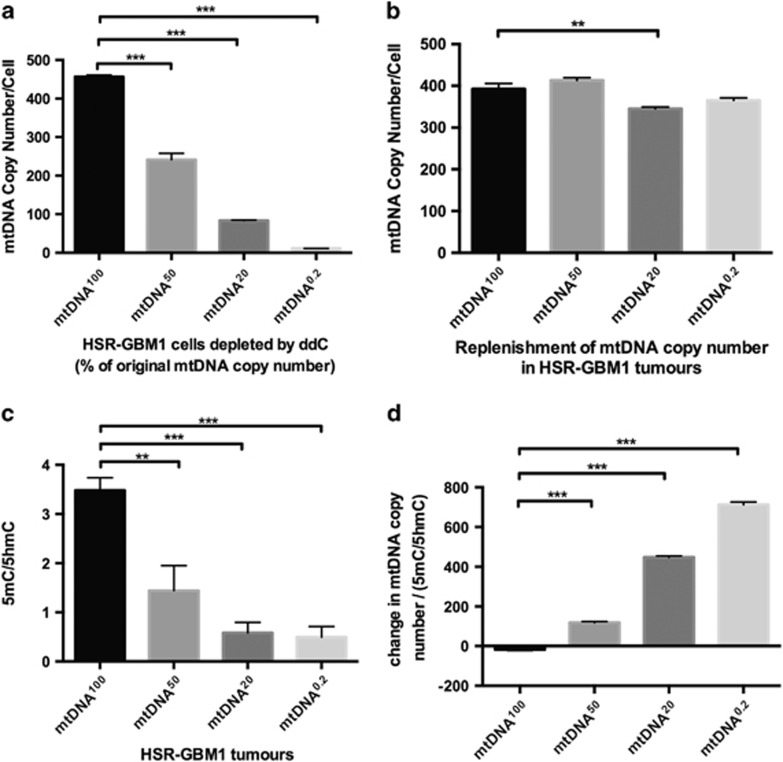
DNA methylation levels at exon 2 of *POLGA* and changes in mtDNA copy number in mtDNA-depleted HSR-GBM1 cells. (**a**) MtDNA copy number in HSR-GBM1 cells that have been depleted to 50%, 20% and 0.2% of their original mtDNA copy number. (**b**) mtDNA copy number replenishment in tumours derived from HSR-GBM1 cells from **a**. (**c**) Enrichment of 5mC/5hmC ratios in all HSR-GBM1 tumours. (**d**) Recovery of mtDNA copy number relative to 5mC/5hmC ratios in all HSR-GBM1 tumours. Significance: **P<0.01; ***P<0.001

To determine if cancer cells can modulate mtDNA copy number and DNA methylation at exon 2 of *POLGA*, we analysed HSR-GBM1 cells that had been depleted to varying degrees of their mtDNA and injected into immunocompromised mice to form tumours^17^ (Figure 2a). Although all tumours replenished their mtDNA to similar levels (Figure 2b), tumours derived from more extensively depleted cells (mtDNA^20^ and mtDNA^0.2^) had higher levels of enrichment for 5hmC than 5mC at exon 2 of *POLGA* (*P*<0.01; Figure 2c). The ratios of mtDNA copy number as a function of enrichment of 5mC:5hmC demonstrate that higher levels of DNA demethylation are correlated with the more aggressive recovery of mtDNA copy number (*P*<0.001; Figure 2d).

### The effects of DNA demethylation at exon 2 of *POLGA* on mtDNA copy number

**Figure 3:**
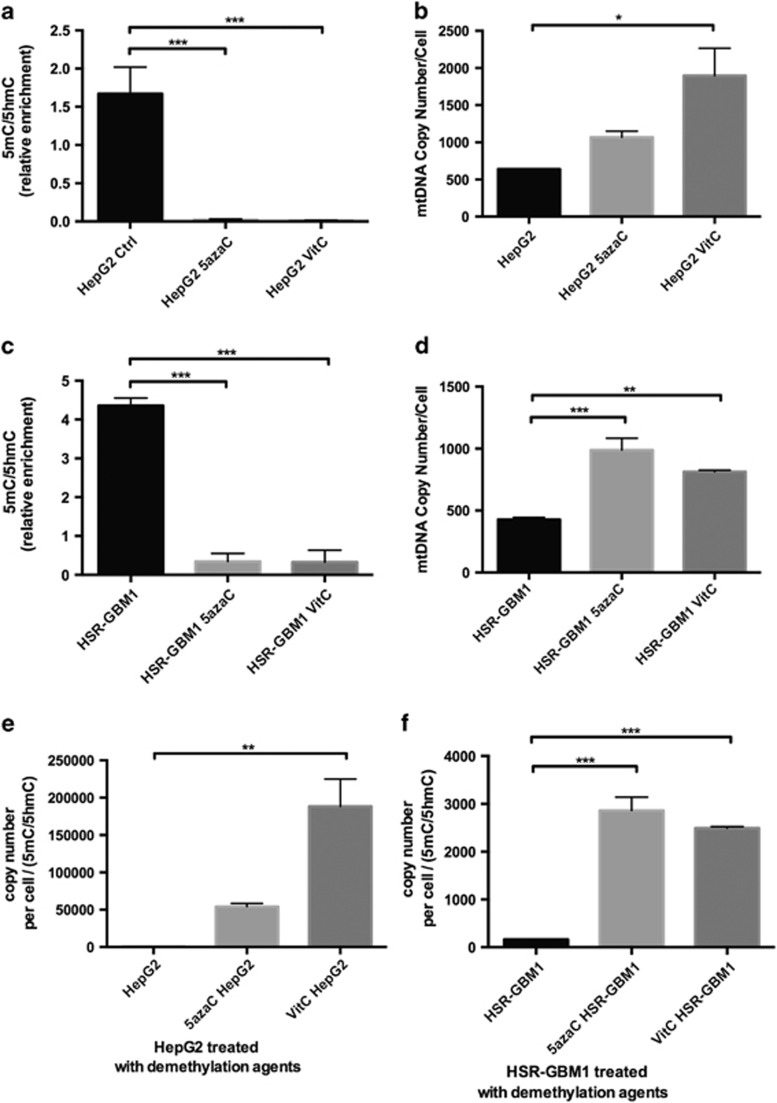
DNA methylation levels at exon 2 of *POLGA* and mtDNA copy number in HepG2 and HSR-GBM1 cells treated with 5-azaC or VitC. (**a**) 5mC/5hmC in HepG2 control (Ctrl) and the two treatment groups. (**b**) mtDNA copy number in HepG2 control and two treatment groups. (**c**) 5mC/5hmC in HSR-GBM1 control and two treatment groups. (**d**) mtDNA copy number in HSR-GBM1 control and two treatment groups. (**e**) mtDNA copy number over ratios of 5mC/5hmC in all groups of HepG2. (**f**) mtDNA copy number over ratios of 5mC/5hmC in all groups of HSR-GBM1. Significance: *P<0.05; **P<0.01; ***P<0.001

To further assess the relationship between DNA methylation at exon 2 of *POLGA* and mtDNA copy number, HepG2 and HSR-GBM1 cancer cells were treated with the global DNA demethylation agents 5-azaC and vitamin C (VitC). Following 48 h of treatment with 5-azaC, enrichment of 5mC significantly decreased in both cell lines at exon 2 of *POLGA,* whereas 5hmC increased (*P*<0.001; Figures 3a and c), indicating active DNA demethylation. Furthermore, both cell lines increased mtDNA copy number by twofold or more (Figures 3b and d). This is further highlighted by the ratios of mtDNA copy number as a function of 5mC:5hmC where the ratios for each treatment for both cell types showed significant increases (*P*<0.01; Figures 3e and f) demonstrating that *de novo* DNA demethylation at exon 2 of *POLGA* promotes replication of mtDNA. Increases in mtDNA copy number were reflected in increases of 1.46 and 1.35-fold in basal O_2_ consumption rates following treatment with 5-azaC and VitC, respectively (Supplementary Table II).

**Figure 4:**
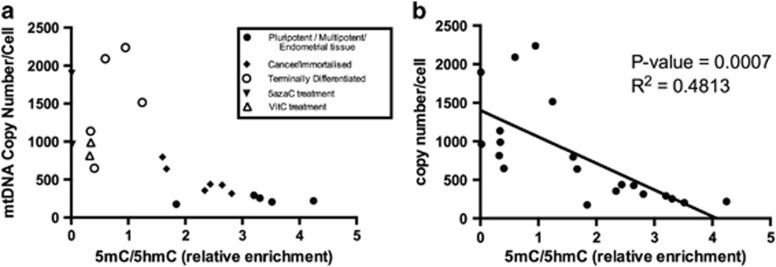
(**a**) Plot of relative enrichment for 5mC/5hmC at exon 2 of *POLGA* and mtDNA copy number in cancer cells, pluripotent and multipotent stem cells, post-mitotic tissues, and HepG2 and HSR-GBM1 cells treated with DNA demethylation agents. (**b**) Linear regression analysis of all the cells analysed in **a**

To determine if there is an overall relationship between mtDNA copy number and 5mC/5hmC at exon 2 of *POLGA*, we merged data sets from Figures 1,2 and 3. Treatment with 5-azaC and VitC produced a 2.0 to 2.5-fold shift in the ratio of mtDNA copy number as a function of 5mC/5hmC (Figure 4a). Consequently, the treated cells clustered with the terminally differentiated somatic cells away from the pluripotent/multipotent and cancer cell populations. Furthermore, linear regression analysis of all cells demonstrated a linear relationship between mtDNA copy number and 5mC/5hmC that is highly statistically significant (*P*=0.0007; Figure 4b).

To determine whether 5-azaC and VitC modulated the intermediates of DNA demethylation, western blot was performed on HepG2 cells. The results showed no change in expression for the DNA methyltransferase, *de novo* methyltransferase 1 (DNMT1), which modulates DNA methylation, for both treatment groups. However, there was a small increase in expression of ten-eleven translocation methylcytosine dioxygenase 1 (TET1), which modulates the conversion of 5mC to 5hmC, for the VitC-treated group when compared with untreated HepG2 cells (Supplementary Figure S2).

### The effects of 5-azaC and VitC on RNA polymerase II activities in *POLGA*

**Figure 5:**
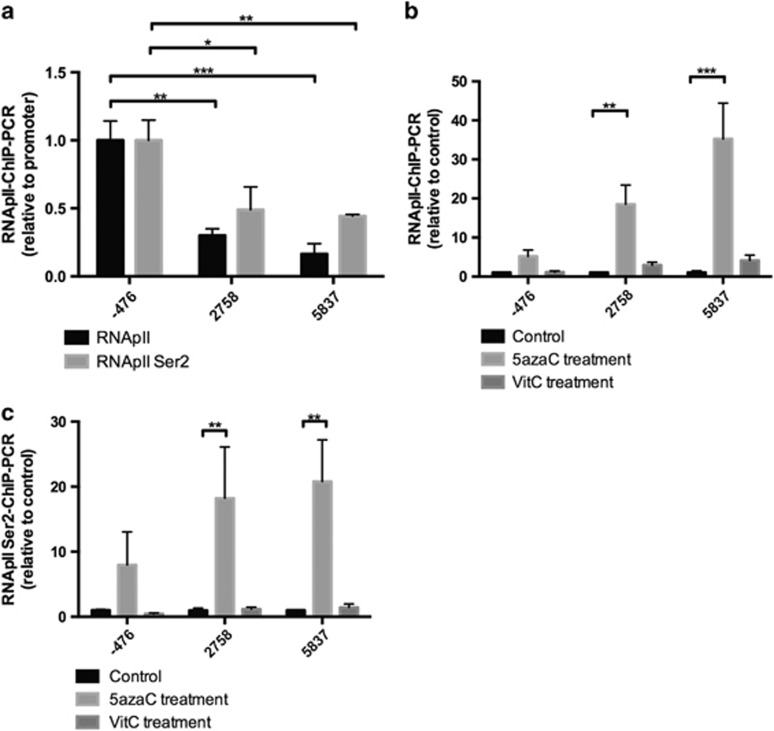
ChIP analysis for enrichment of RNApII and RNApII Serine 2 in (**a**) untreated HSR-GBM1 cells. (**b**) ChIP analysis for RNApII in control HSR-GBM1 and HSR-GBM1 cells treated with 5-azaC (48 h) and VitC (72 h). (**c**) ChIP analysis for RNApII Serine 2 in control HSR-GBM1 and HSR-GBM1 cells treated with 5-azaC and VitC. The numbers represent the loci distance from the transcriptional start site (TSS) of *POLGA*. Significance: *P<0.05; **P<0.01; ***P<0.001

As DNA methylation regulates transcript levels by altering chromatin structure and regulating the elongation by RNA polymerase II (RNApII)^36, 37, 38^ and through the human transcription elongation regulator interacting with RNApII at the Serine 2 position (RNApIISer2),^39^ we determined whether treatment with 5-azaC and VitC modulates DNA methylation at exon 2 of POLGA and thus RNApII function. Using chromatin immunoprecipitation (ChIP), we quantified the enrichment of RNApII and the elongating RNApIISer2 in HSR-GBM1 cells. There was reduced enrichment of RNApII and RNApII phosphorylated on Ser2 in two regions downstream of the CpG islands in exon 2 when compared with the promoter region that is upstream of exon 2 of *POLGA* (*P*<0.05; Figure 5a). This indicates that hypermethylation at exon 2 of *POLGA* suppresses RNApII activities and its elongation to low levels. HSR-GBM1 cells treated with 5-azaC significantly increased RNApII activities in the two regions downstream of the CpG islands in exon 2 (*P*<0.01; Figure 5b) and increased RNApIISer2 (*P*<0.01; Figure 5c), suggesting that demethylation at exon 2 allows downstream RNApII activities within *POLGA*. However, VitC did not induce a significant change in enrichment for both RNApII (Figure 5b) and RNApII Ser2 (Figure 5c).

### Cancer cells require a DNA demethylation agent to progressively increase mtDNA copy number during differentiation

**Figure 6:**
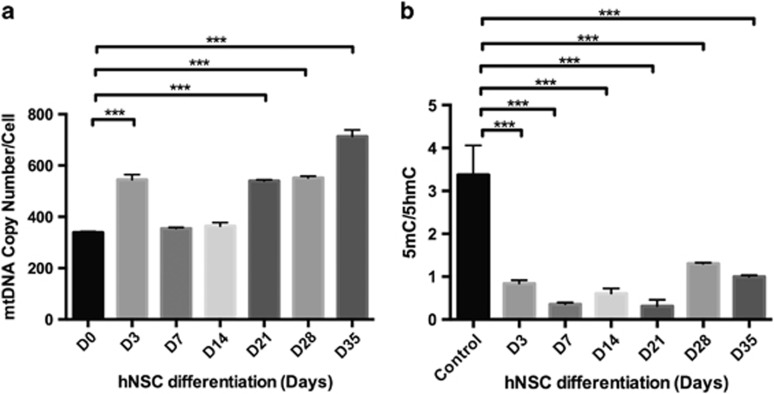
hNSCs induced to differentiate towards astrocytes over 35 days. (**a**) MtDNA copy number. (**b**) 5mC/5hmC ratios. (D=day). Significance: ***P<0.001

It has been previously shown that human stem cells increase mtDNA copy number as they undergo differentiation into mature cells that have higher demands for OXPHOS-derived ATP, such as hNSCs differentiating into astrocytes.^17^ Here, we show that hNSCs modulate mtDNA copy number (Figure 6a) through significant changes to the ratios of 5mC and 5hmC at exon 2 of *POLGA* during astrocyte differentiation (Figure 6b). However, as previously shown, HSR-GBM1 cells failed to increase mtDNA copy number and did not maintain elevated expression of the astrocyte end point marker, GFAP, as they were induced to differentiate into astrocytes.^17^ As HSR-GBM1 cells increase mtDNA copy number under treatment with global DNA demethylating agents (Figures 3a and f), we investigated whether this treatment induces a synchronous increase in mtDNA copy number and loss of expression of multipotent gene markers during astrocyte differentiation, which untreated HSR-GBM1 cells fail to achieve.

**Figure 7:**
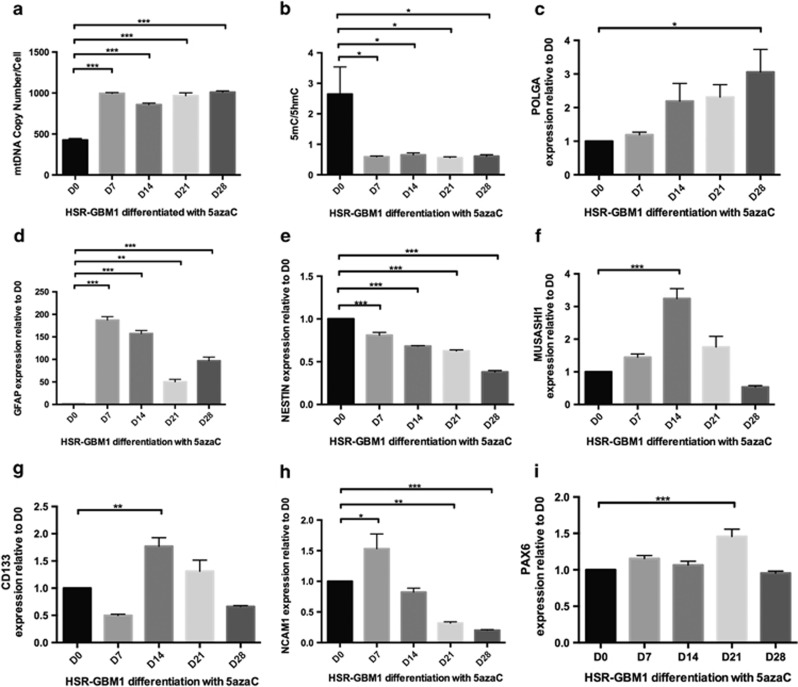
HSR-GBM1 cells induced to differentiate towards astrocytes with treatment of 5-azaC over 28 days. (**a**) mtDNA copy number; (**b**) DNA methylation levels (5mC/5hmC) at exon 2 of *POLGA*; gene expression for (**c**) *POLGA*; (**d**) *GFAP*; (**e**) *NESTIN*; (**f**) *MUSASHI1*; (**g**) *CD133*; (**h**) *NCAM1*; and (**i**) *PAX6* (D=day). Significance: *P<0.05; **P<0.01; ***P<0.001

Following the induction of HSR-GBM1 cells into astrocytes with continuous treatment with 5-azaC or VitC, there was a twofold increase in mtDNA copy number for both 5-azaC-treated and VitC-treated HSR-GBM1-derived astrocytes on day 7 (*P*<0.001; Figure 7a; Supplementary Figure S3A). We further assessed basal O_2_ consumption rates on day 7, which increased by 1.7-fold compared with equivalent time point controls (Supplementary Table II). However, mtDNA copy number remained consistently high in 5-azaC-treated cells (*P*<0.001) whereas, for the VitC-treated cells, mtDNA copy number decreased over 28 days (*P*<0.05; Supplementary Figure 3A).

The levels of DNA demethylation at exon 2 of *POLGA* also increased under both treatments (*P*<0.05; Figure 7b; Supplementary Figure 3B). Yet, unlike 5-azaC-treated cells that maintained low levels of DNA methylation, VitC-treated HSR-GBM1 cells re-established DNA methylation after day 14 (Supplementary Figure 3B) and decreased mtDNA copy number. The increases in mtDNA copy number and demethylation at exon 2 following 5-azaC treatment were matched by increased expression of *POLGA* (*P*<0.05; Figure 7c). However, loss of mtDNA copy number after an initial increase, and increasing DNA methylation at exon 2, were matched by loss of *POLGA* expression for the VitC-treated group (Supplementary Figure 3C).

Both 5-azaC and VitC induced elevated levels of expression for *GFAP* over 28 days of differentiation (*P*<0.001; Figure 7d; Supplementary Figure 3D). However, the neural stem cell markers showed variable patterns of gene expression. Although expression of *NESTIN* progressively decreased during differentiation following both treatments (*P*<0.001; Figure 7e, Supplementary Figure 3E), expression of *MUSASHI1*, (Figure 7f; Supplementary Figure 3F), *CD133* (Figure 7g; Supplementary Figure 3G) and *NCAM* (Figure 7h; Supplementary Figure 3H) decreased following 5-azaC treatment but rose during VitC treatment. However, *PAX6* remained at similar levels by day 28 for the 5-azaC cohort (Figure 7i) and rose during VitC treatment (Supplementary Figure 3I).

### Rebound effects on VitC-treated HSR-GBM1

**Figure 8:**
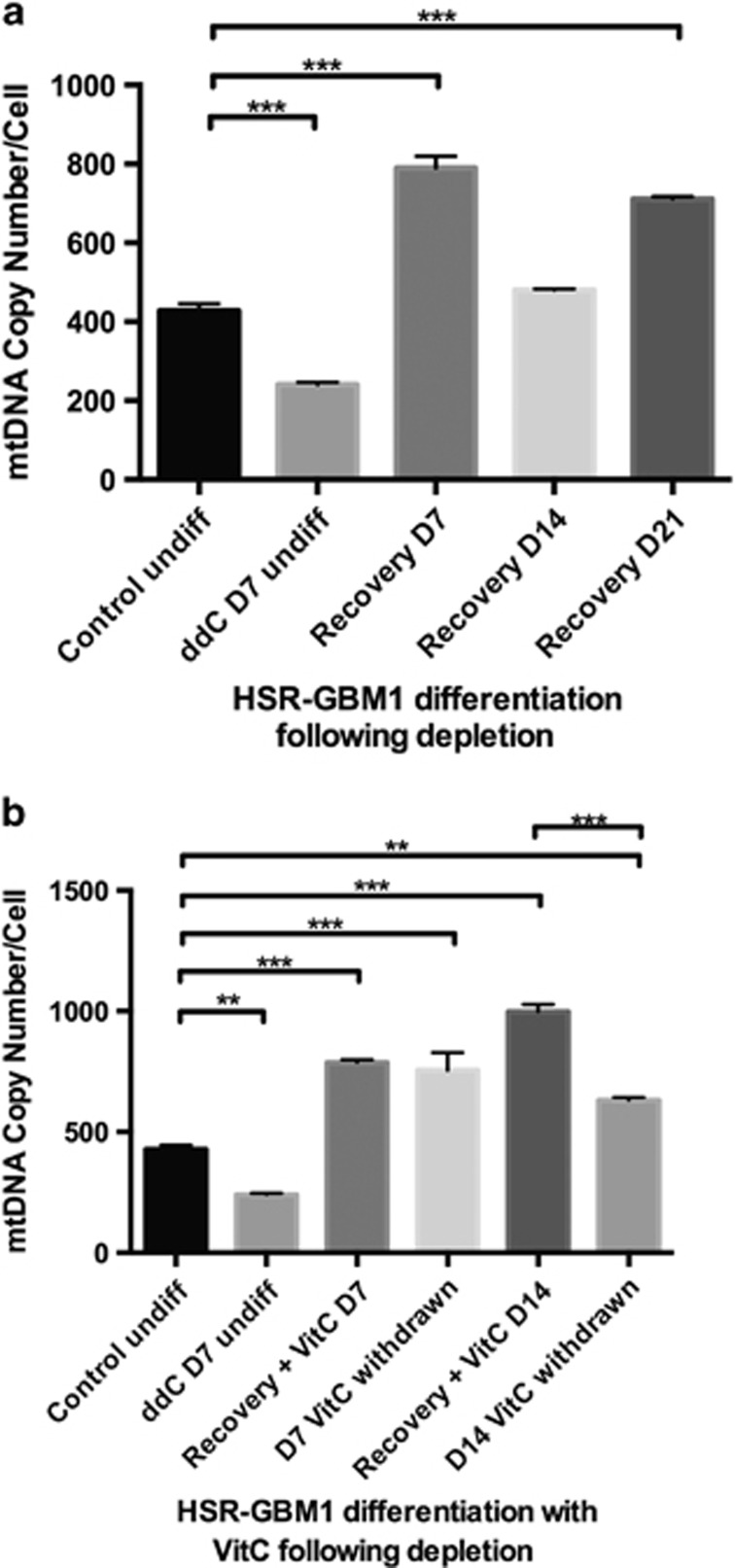
MtDNA copy number in HSR-GBM1 cells, which were treated with ddC to deplete mtDNA for 7 days and were recovered in astrocyte induction media (**a**) for 21 days; (**b**) for 14 days with VitC treatment and another 7 days post-withdrawal of VitC. (D=day). Significance: **P<0.01; ***P<0.001

We investigated whether modification of mtDNA copy number before differentiation would enhance differentiation in cancer cells. HSR-GBM1 cells were first treated with the mtDNA depletion agent, 2′,3′-dideoxycytidine (ddC) for 7 days to reduce mtDNA copy number by approximately 50%. The cells were then induced to differentiate into astrocytes for 7 and 14 days. MtDNA copy number increased fourfold following depletion after 7 days of recovery (*P*<0.001; Figure 8a). Although HSR-GBM1 cells maintained levels above non-depleted cells after 14 and 21 days of recovery, they did not surpass levels for 7 days of recovery. Depleted cells were also induced to differentiate into astrocytes in the presence of VitC for 7 and 14 days (Figure 8b). This resulted in the continued increase in mtDNA copy number at day 14, which was not observed in cells recovering in the absence of VitC for 14 days (*P*<0.001). These findings demonstrate that mtDNA copy number can be reset in cancer cells, which results in the expansion of mtDNA copy number during differentiation. However, this is only maintained in the presence of a DNA demethylation agent.

## Discussion

Pluripotent cells, such as ESCs, establish the mtDNA set point, which is defined as maintaining low mtDNA copy number in the presence of the OCT4-SOX2-NANOG network.^6, 7, 13^ As a result, they utilise glycolysis,^40, 41^ which promotes self-renewal and a highly proliferative state. Glycolysis is also used in zygotes and preimplantation embryos, suggesting that ESCs rely on the same energy-generating pathway as their precursor cells.^42, 43, 44^ ESCs can increase mtDNA copy number in a cell-specific manner during differentiation ensuring that specialised cells acquire the appropriate number of mtDNA copies to meet their specific requirements for OXPHOS-derived ATP.^6^ Cancer cells have low mtDNA copy number and rely predominantly on aerobic glycolysis instead of OXPHOS, even under normoxic conditions, which also promotes a highly proliferative state.^21, 22^

We show that the CpG islands in exon 2 of *POLGA* were highly methylated in cancer, pluripotent and multipotent cells. As DNA methylation inhibits downstream transcription of *POLGA*, high levels of DNA methylation are associated with low levels of *POLGA* expression and low mtDNA copy number in these highly proliferative cells. However, pluripotent and multipotent cells are capable of differentiating and synchronously replicating mtDNA. As a result, terminally differentiated cells exhibit higher mtDNA copy number and reduced levels of DNA demethylation within *POLGA* in a tissue-specific manner. A similar state exists in mouse cells except that exon 2 of *PolgA* undergoes extensive DNA methylation in order for *PolgA* to be expressed.^9^ Nevertheless, cancer cells are unable to modulate DNA methylation at this location and fail to differentiate.^17^ Interestingly though, we further demonstrate that mtDNA-depleted cancer cells can modulate DNA demethylation at exon 2 of *POLGA* to enable replenishment of mtDNA to pre-depleted levels, which suggests that each tumour cell type has a defined number of mtDNA copies or set point. It appears that this modulation is essential to tumour formation in immunocompromised mice^17^ with all tumours formed from HSR-GBM1 mtDNA-depleted cells acquiring similar mtDNA copy number.

Short-term treatment of cancer cells with 5-azaC significantly increased DNA demethylation of the CpG islands in exon 2 of *POLGA* leading to upregulation of RNA polymerase activities, transcriptional elongation, and increases in mtDNA copy number and basal O_2_ consumption rates. 5-azaC acts by incorporating into DNA that is covalently bound to DNMT1, a maintenance methyltransferase, to reduce its enzymatic activity and modulate global demethylation of chromosomal DNA.^25^ However, this does not reduce DNMT1 protein levels, which resulted in DNA methylation being reconstituted once 5-azaC is withdrawn. Extension of 5-azaC treatment to 28 days during differentiation produced continued demethylation at exon 2 of *POLGA* and reduced expression of early markers of neural differentiation. This coincided with significant increases in mtDNA copy number and *GFAP* gene expression in a similar manner to differentiating hNSCs.^17^ Consequently, long-term modulation of global DNA methylation, which also acts at exon 2 of *POLGA*, induces differentiation by synchronously reducing multipotent gene expression while increasing mtDNA copy number and endpoint gene expression.

VitC, on the other hand, does not inhibit DNMT1. It enhances the enzymatic activity of TET1 by increasing the activity of Fe(II) 2-oxoglutarate dioxygenase within the catalytic domain of TET1 and converting 5mC to 5hmC.^45^ Although this induces global demethylation, analysis of 5mC and 5hmC enrichment and mtDNA copy number during 28 days of differentiation of cancer cells demonstrates reversible changes that are indicative of the ‘rebound effect'.^30^ Moreover, increased expression of early neural markers suggests that these cells failed to fully differentiate into astrocytes. Similarly, when cancer cells were depleted of mtDNA and induced to differentiate in the presence of VitC, they increased mtDNA copy number on days 7 and 14, which suggests the establishment of a mtDNA set point associated with the capability to differentiate. However, there was an immediate reduction in mtDNA copy number over the next 7 days post-withdrawal of VitC, which results from the inability of VitC to inactivate DNMT1.

Overall, we have demonstrated a correlation between decreased DNA methylation levels at exon 2 in human *POLGA* and increased mtDNA copy number. Pluripotent and multipotent cells possessed high levels of DNA methylation at exon 2 of *POLGA*. This restricts the expression of *POLGA*, which, at low levels, suppresses mtDNA replication and results in low mtDNA copy number. Similarly, cancer cells were hypermethylated at exon 2 of *POLGA* and exhibited low mtDNA copy number. Pluripotent and multipotent cells were able to increase mtDNA copy number during differentiation and *POLGA* is demethylated at exon 2. However, cancer cells can only overturn the hypermethylated state in the presence of demethylation agents, such as 5-azaC, which promotes mtDNA replication to high levels observed in terminally differentiated cells. This state is only maintained in the presence of a DNA demethylation agent that inhibits DNMT1 interaction at exon 2 of *POLGA*, otherwise cells reduce mtDNA copy number and return to a more dedifferentiated state.

## Materials and Methods

### Cell culture

The human Glioblastoma multiforme cell line HSR-GBM1 and hNSCs derived from the NIH-approved human ESC line H9 (WA09; Invitrogen Life Technologies, Carlsbad, CA, USA) were cultured in StemPro Complete Media consisting of Dulbecco's modified essential medium/F12 (1 : 1 DMEM/F12; Invitrogen, Mulgrave, VIC, Australia), 2% StemPro neural supplement (Invitrogen Life Technologies), epidermal growth factor (Millipore, Billerica, MA, USA) and fibroblast growth factor (FGF; Millipore) at 37 °C with 5% CO_2_ and 95% humidity.

Cells from the human multiple myeloma (U266) cell line were cultured in RPMI 1640 medium (Invitrogen), 10% foetal bovine serum (FBS; Gibco Life Technologies, Mulgrave, VIC, Australia), 1% GlutaMax (Invitrogen) and 1% sodium pyruvate (Gibco Life Technologies) at 37 °C with 5% CO_2_.

Human heptocellular carcinoma (HepG2) cells, HDFa human dermal fibroblasts and BJ fibroblasts were cultured in DMEM (Invitrogen) with 10% FBS, 1% GlutaMax and 0.5% penicillin/streptomycin (Invitrogen) at 37 °C with 5% CO_2_.

The human ovarian carcinoma (SK-OV-3) cell line was cultured in ATCC-formulated McCoy's 5A Medium (Invitrogen) and 10% FBS at 37 °C with 5% CO_2_.

The hESC line MEL-1 was routinely cultured on a feeder layer of inactivated mouse embryonic fibroblasts in DMEM plus 20% FBS (HES media) with passaging every 6–7 days. For differentiation, small areas of undifferentiated cells were mechanically excised from day 7 colonies and placed in low attachment wells with HES media. Resulting embryoid bodies (EBs) were cultured in DMEM/F12 plus 20% serum replacement. At day 5, EBs were transferred into neural media consisting of DMEM/F12 with 1% N2 (Gibco Life Technologies), 2 *μ*g/ml Heparin and 20 ng/ml bFGF and left until day 7 when EBs were plated out to Ornithine/Laminin (20 and 10 *μ*g/ml, respectively) coated dishes in neural media. EBs attached and formed outgrowths, which were monitored for the formation of neural rosettes. Rosettes were mechanically excised at day 13 and placed back into low attachment wells in neural media and the resultant neurospheres remained in suspension culture until day 22. Samples were collected for analysis at day 13 (rosette) and day 22 (neurosphere).

Breast, placental and endometrial tissues and MCF7 breast cancer and MCF10a breast epithelial cell lines were kindly provided by Dr. Kristy Brown, Dr. Stefan White and Associate Professor Caroline Gargett under human research ethics numbers 109B, 11281B and 09057B Southern Health HREC, respectively. Normal human brain samples were obtained from the Victorian Brain Bank Network under the human research ethics number 09023B, Southern Health, HREC.

### Differentiation assays

Undifferentiated HSR-GBM1 cells and hNSCs were seeded in six-well plates coated with 20 *μ*g/ml of fibronectin at a density of 2.5 × 10^4^ cells/cm^2^ with astrocyte induction media consisting of DMEM/F12, 1% N2 supplement (Gibco Life Technologies) and 2% FBS. They were cultured at 37 °C with 5% CO_2_.

### DNA demethylation treatment

HSR-GBM1 and HepG2 cells were cultured in the presence of 5-azaC (Sigma-Aldrich, Castle Hill, NSW, Australia) or VitC (L-ascorbic acid, Sigma). Cultures were treated daily with 0.5 *μ*M 5-azaC or 100 *μ*g/ml VitC. 5-azaC and VitC-treated cultures were harvested after 48 and 72 h, respectively. For further culture to 7, 14, 21 and 28 days, see Differentiation assays.

### DNA and RNA extraction

Total DNA and RNA were extracted using the DNeasy Blood and Tissue Kit (Qiagen, Valencia, CA, USA) and RNeasy Mini Kit (Qiagen), respectively, as described in Dickinson *et al.*^17^ The DNA samples were treated with RNase solution (Qiagen) and Proteinase K Solution (Qiagen) at 65 °C for 10 min and RNA samples were treated with DNase I (Qiagen) for 20 min.

### Gene expression analysis

cDNA was synthesised, according to the manufacturer's instructions, using the Superscript III First-Strand Synthesis System (Life Technologies, Mulgrave, VIC, Australia). PCR products were generated by RT-PCR using the Rotorgene-3000 real-time PCR machine (Qiagen Corbett Research, Cambridge, UK) under specific primer conditions (Supplementary Table SI).^17^ *β*-Actin, GAPDH and OAZ1 (Ornithine Decarboxylase Antizyme 1) were tested as house keeping genes and OAZ1 was chosen to normalise and analyse the levels of gene expression as it is regarded to be the more stable housekeeper for drug treatment.^46^

### Bisulphite sequencing

One microgram of extracted DNA was incubated with 3 M NaOH for 10 min at 37 °C and bisulphite treated using the MethylEasy Xceed Kit (Human Genetics Signatures, North Ryde, NSW, Australia). The bisulphite-treated samples were amplified using nested PCR primers (Supplementary Table SI). PCR products were ligated into a pCR 2.1 Vector (Invitrogen) and transformed into DH5*α* competent cells (Invitrogen). Colonies containing individual clones were amplified using M13 primers (Supplementary Table SI) and sequenced using a 16-capillary 3130xi Genetic Analyzer (Applied Biosystems, Mulgrave, VIC, Australia). BiQ Analyzer (http://biqanalyzer.bioinf.mpi-inf.mpg.de/) was used to assess the extent of DNA methylation.

### Real-time PCR for mtDNA copy number

Real-time PCR (quantitative PCR, qPCR) was performed on purified total DNA using the Rotor-Gene 3000. A total volume of 20 *μ*l of mixture included 10 *μ*l SensiMix SYBR, 1 *μ*l of each forward and reverse primer at a concentration of 5 *μ*M, 6 *μ*l of sterile ddH_2_O and 2 *μ*l of the DNA of interest. Primer sequences and reaction conditions are listed in Supplementary Table SI. Melt curve analysis was performed before each reaction to determine melting temperatures for the second acquisition phase, which was between 75 and 82 °C for all primers.

### Immunoprecipitation of methylated DNA

MeDIP was performed, as previously described.^47^ Extracted DNA was sonicated to fragments sized between 200 and 1000 bp. Three microgram of the sonicated DNA was denatured at 95 °C for 10 min and immunoprecipitated with 2 *μ*g of antibodies to either 5mC (Active Motif, Carlsbad, CA, USA) or 5hmC (Active Motif) with 20 *μ*l of Protein G Dynabeads (Invitrogen) in 500 *μ*l of IP buffer (10 mM Na-phosphate, pH 7.0, 140 mM NaCl, 0.05% Triton X-100) at 4 °C overnight. Samples were washed three times with 700 *μ*l IP buffer and DNA was eluted using 250 *μ*l of Proteinase K digestion buffer (5 mM Tris, 1 mM EDTA, pH8.0, 0.05% SDS) supplemented with 7 *μ*l of Proteinase K (20 *μ*g/*μ*l; Qiagen) at 50 °C for 3 h. The immunoprecipitated DNA was then purified using phenol–chloroform purification and precipitated with 3 *μ*g of glycogen (Invitrogen) and 100% ethanol at −80 °C for a minimum of 2 h.

### MeDIP-qPCR analysis

qPCR was performed on the MeDIP samples using the Rotorgene-3000 machine under specific primer conditions (Supplementary Table SI). The data were normalised using the percentage input method to determine the levels of enrichment for 5mC and 5hmC within the nuclear-encoded *POLGA*. Fold changes between 5mC and 5hmC were further determined.

### Chromatin immunoprecipitation

ChIP was performed, as previously described.^9^ Cells were cross-linked with 1% formaldehyde for 10 min and quenched with 125 mM glycine (Sigma-Aldrich) for 5 min. Cross-linked samples were lysed at 4 °C for 10 min and sonicated to generate chromatin fragments with an average size of 200–800 bp. Chromatin from 1 × 10^6^ cells was immunoprecipitated with Protein G Dynabeads and anti-RNApII (clone 4H8) and anti-Ser2 RNApII antibodies (both from Abcam, Waterloo, NSW, Australia). Cross-links in immunoprecipitated samples were reversed by incubating with 200 mM NaCl and 10 mg/ml of Proteinase K at 65 °C for 16 h and purified using the Qiagen PCR purification kit. Samples were analysed using qPCR with primers described in Supplementary Table SI.

### Western blotting

Cells were lysed in a buffer containing 150 mM NaCl, 50 mM Tris (pH 7.4), 1 mM EDTA, 0.5% Triton X-100, protease inhibitor cocktail (Sigma-Aldrich), 1 mM NaF, 1 mM *β*-glycerophosphate, 1 mM Na_3_VO_4_ and 1 mM DTT. Protein preparations were resolved by 8% SDS-PAGE, transferred onto PVDF membranes (Millipore), and the membranes were incubated for 1 h in Blocking Buffer (Li-Cor, Lincoln, NE, USA). Blots were probed with primary antibodies for DNMT1 and TET1 (both from Abcam) diluted in blocking buffer (Li-Cor) overnight at 4 °C. The blots were washed and incubated with 1 : 15 000 dilution of fluor-conjugated secondary antibody (Li-Cor) and scanned using an Odyssey infrared scanner (Li-Cor).

## Abbreviations

mtDNA: mitochondrial DNA
5mC: 5-methylcytosine
5hmC: 5-hydroxymethylcytosine
POLGA: DNA polymerase gamma A
5-azaC: 5-azacytidine
VitC: vitamin C
OXPHOS: oxidative phosphorylation
hESC: human embryonic stem cell
hNSC: human neural stem cell
GFAP: glial fibrillary acidic protein
HSR-GBM1: human glioblastoma multiforme cells
U266: human myeloma cells
HepG2: human heptocellular carcinoma cells
SK-OV3: human ovarian carcinoma cells
MEL-1: human embryonic stem cells
EB: embryoid body
HDFa: human dermal fibroblast
BJ: human skin fibroblast
MCF7: human breast adenocarcinoma cell line
MCF10a: human mammary epithelial cells
qPCR: quantitative PCR
MeDIP: immunoprecipitation of methylated DNA
ChIP: chromatin immunoprecipitation
RNApII: RNA polymerase II
RNApIISer2: RNA polymerase II at Serine 2
DNMT1: *de novo* methyltransferase 1
TET1: ten-eleven translocation methylcytosine dioxygenase 1
ddC: 2′,3′-dideoxycytidine
