# Supplementary figures and images for: Mitochondrial DNA copy number is regulated by DNA methylation and demethylation of POLGA in stem and cancer cells and their differentiated progeny
Source: Cell Death Dis. 2015 Feb 26;6(2):e1664–. doi: 10.1038/cddis.2015.34 (PMC4669800; doi:10.1038/cddis.2015.34)

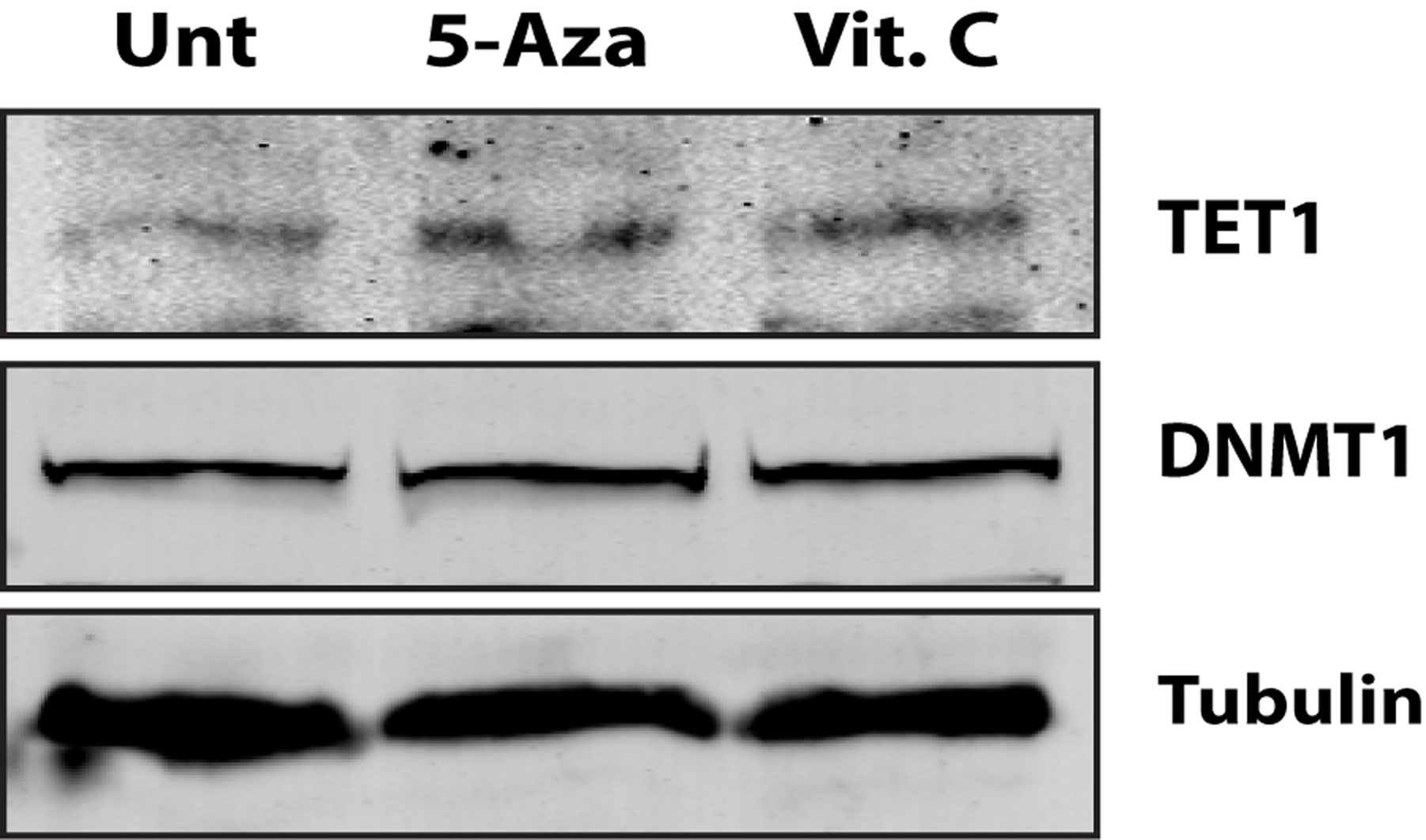

Supplement: Supplementary Figure S2 [file cddis201534x2.tif]

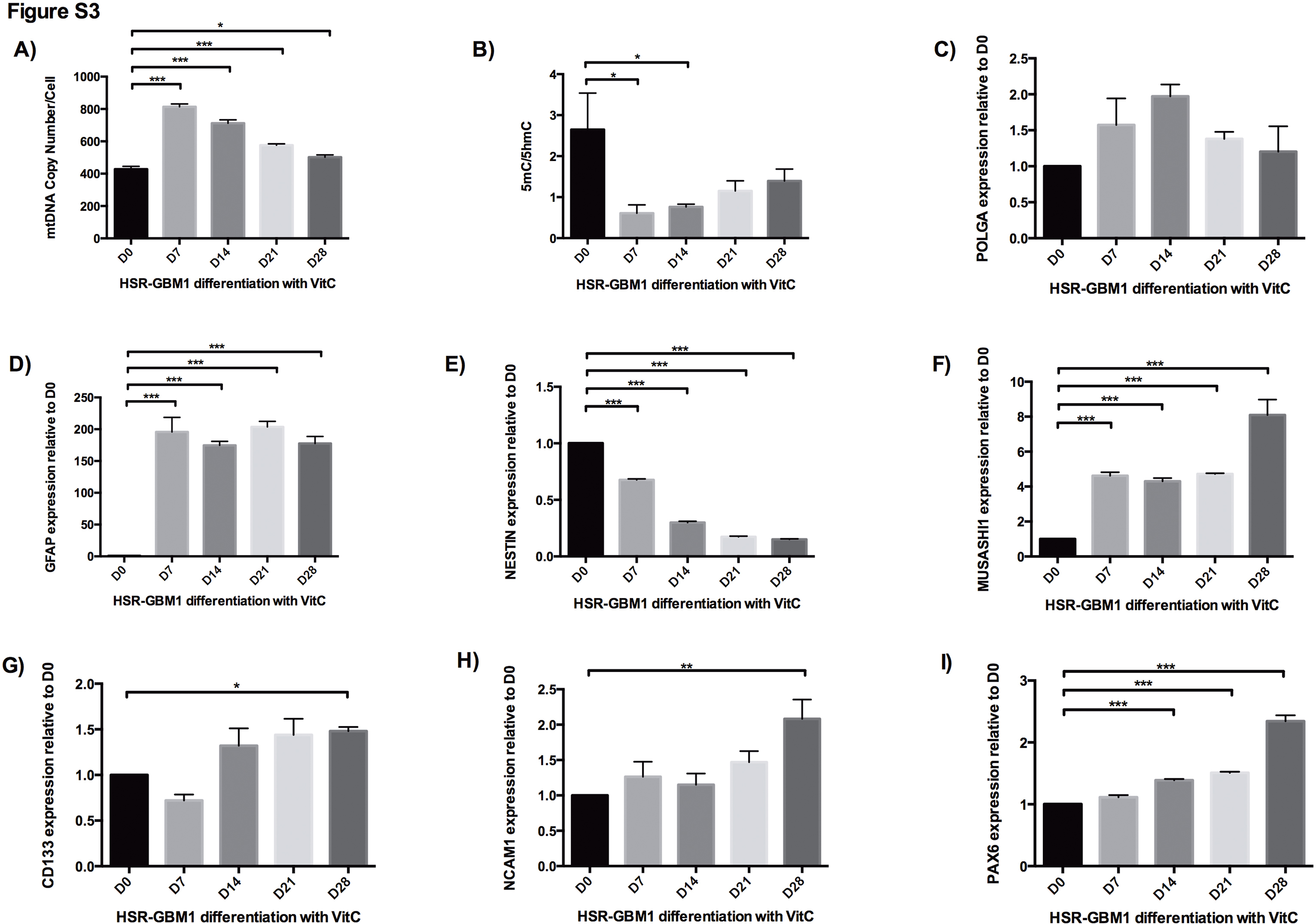

Supplement: Supplementary Figure S3 [file cddis201534x3.tif]
