# Supplementary material for: Mitochondrial DNA copy number is regulated by DNA methylation and demethylation of POLGA in stem and cancer cells and their differentiated progeny
Source: Cell Death Dis. 2015 Feb 26;6(2):e1664–. doi: 10.1038/cddis.2015.34 (PMC4669800; doi:10.1038/cddis.2015.34)
Supplement: Supplementary Table S1 [file cddis201534x4.doc]

| **Table S1** | | | |
| --- | --- | --- | --- |
| **mtDNA copy number** | |  | **product size** |
| human Beta-Globin | hBeta-Globin-F | CAACTTCATCCACGTTCACC | 268 |
|  | hBeta-Globin-R | GAAGAGCCAAGGACAGGTAC |  |
| human mtDNA | h-mtDNA-F | CGAAAGGACAAGAGAAATAAGG | 152 |
|  | h-mtDNA-R | CTGTAAAGTTTTAAGTTTTATGCG |  |
| **MeDIP** | | | |
| POLG EXON2 | hPolg Exon2-F | CAGACCTCCACGTCGAACAC | 162 |
|  | hPolg Exon2-R | GACAACCTGGACCAGCACTT |  |
| BETA-GLOBIN | hBeta-Globin-F | CAACTTCATCCACGTTCACC | 170 |
|  | hBeta-Globin-R | GAAGAGCCAAGGACAGGTAC |  |
| COXII | hCox2-F | CTGCTTCCTAGTCCTGTATG | 235 |
|  | hCox2-R | GTCGGTGTACTCGTAGGTTC |  |
| CYTB | hCytB-F | ATGACCCCAATACGCAAAACT | 401 |
|  | hCytB-R | GGGAGGACATAGCCTATGAA |  |
| ND6 | hND6-F | CCGCACCAATAGGATCCTCCCGA | 187 |
|  | hND6-R | GCATGGGGGTCAGGGGTTGAG |  |
| ND1 | hND1-F | TCTCACCATCGCTCTTCTAC | 350 |
|  | hND1-R | GGTTGGTCTCTGCTAGTGTG |  |
| ATP6 | hATP6-F | CAGTGATTATAGGCTTTCGCTC | 343 |
|  | hATP6-R | GTGTTGTCGTGCAGGTAGAG |  |
| **Bisulphite Sequencing** | | | |
| Polg Exon2 primary | hPolg Exon2-1F | CAAGTTCCCTCTGCCAAGCA | 315 |
|  | hPolg Exon2-1R | CGTCGAGCACCTGCAGAA |  |
| Polg Exon2 Nested | hPolg Exon2-F | CAGACCTCCACGTCGAACAC | 162 |
|  | hPolg Exon2-R | GACAACCTGGACCAGCACTT |  |
| M13 | M13-R | CTGGCCGTCGTTTTAC |  |
|  | M13-R | CAGGAAACAGCTATGAC |  |
| **Gene Expression** | | | |
| OAZ1 | hOAZ1-F | GGATCCTCAATAGCCACTGC | 150 |
|  | hOAZ1-R | TACAGCAGTGGAGGGAGACC |  |
| NESTIN | hNestin-F | AAACCAGAGCCATGAGACAC | 156 |
|  | hNestin-R | TGGCCTACAGCCTCTTTTTC |  |
| MUSASHI1 | hMusashi1-F | AGAAAGCTCAGCCAAAGGAG | 194 |
|  | hMusashi1-R | GAATTCGGGGAACTGGTAGG |  |
| CD133 | hCD133-F | CAGAGTACAACGCCAAACCA | 245 |
|  | hCD133-R | AAATCACGATGAGGGTCAGC |  |
| NCAM1 | hNCAM1-F | GTCATTGTGAATGTGCCACC | 459 |
|  | hNCAM1-R | GTGCCCATCCAGAGTCTTTT |  |
| PAX6 | hPAX6-F | TTTAAACTCTGGGGCAGGTC | 198 |
|  | hPAX6-R | GGAGTTGCTGGTGAGAGTTT |  |
| GFAP | hGFAP-F | GAAGCTCCAGGATGAAACCA | 165 |
|  | hGFAP-R | ACCTCCTCCTCGTGGATCTT |  |
| POLG | hPOLG-F | CACACCTAAACTCATGGCAC | 436 |
|  | hPOLG-R | GTCCACGTCGTTGTAAGGTC |  |
| **ChIP** | | | |
| -478 | hPolgPrm-F | TCCTCCCTCAGGAACGAAGT | 210 |
|  | hPolgPrm-R | CTCCACTTTCCTCCACCTGC |  |
| 2758 | hPolgChip-F | TAAGCAATGCCCCGAGTACC | 215 |
|  | hPolgChip-R | AGGCTTTGGTGGCTGAAAGA |  |
| 5837 | hPolgEnd-F | AACCAGAGCAAGGGACATGG | 199 |
|  | hPolgEnd-R | TCTGTGGATAGCAGCCAAGC |  |
