# Supplementary material for: Mitochondrial DNA copy number is regulated by DNA methylation and demethylation of POLGA in stem and cancer cells and their differentiated progeny
Source: Cell Death Dis. 2015 Feb 26;6(2):e1664–. doi: 10.1038/cddis.2015.34 (PMC4669800; doi:10.1038/cddis.2015.34)
Supplement: Supplementary Table S2 [file cddis201534x5.doc]

| **Table S2** | | |
| --- | --- | --- |
| **Treatment** | **Basal O2 Consumption**  **(pmol/per sec/10^6^ cells)**  **± SEM** | **ANOVA** |
| **HSR-GBM1-CTL-undifferentiated** | 15.82 ± 0.5638 | a |
| **HSR-GBM1-5AzaC-undifferentiated** | 23.11 ± 0.9665 | b*** |
| **HSR-GBM1-VitC-undifferentiated** | 21.3 ± 0.4429 | b** |
| **HSR-GBM1-CTL-7 day differentiation** | 13.92 ± 0.7754 | a |
| **HSR-GBM1-5AzaC-7 day differentiation** | 23.72 ± 0.7016 | b*** |
