# Supplementary material for: Mitochondrial DNA copy number is regulated by DNA methylation and demethylation of POLGA in stem and cancer cells and their differentiated progeny
Source: Cell Death Dis. 2015 Feb 26;6(2):e1664–. doi: 10.1038/cddis.2015.34 (PMC4669800; doi:10.1038/cddis.2015.34)
Supplement: Supplementary Figure and Table Legends [file cddis201534x6.doc]

**Supplementary Data**

**Supplemental Figure S1.** Bisulphite sequencing on (A) HSR-GBM1 (n = 8); (B) U266 (n = 16); (C) Mel-1 (n = 13); (D) hNSC (n = 9) cells; (E) bisulphite sequencing results displayed as a percentage of DNA methylated sites; P > 0.05.

**Supplemental Figure S2.** Western blotting detecting TET1 and DNMT1 in HepG2 control (Unt) and 5-azaC and VitC treatment groups. TUBULIN is used as a loading control. The images are representative of gels that were each run six separate occasions.

**Supplemental Figure S3.** HSR-GBM1 cells induced to differentiate towards astrocytes with treatment of VitC over 28 days. (A) MtDNA copy number; (B) DNA methylation levels (5mC/5hmC) in exon 2 of *POLGA*; Gene expression for (C) *POLGA* (D) *GFAP*; (E) *NESTIN*; (F) *MUSASHI1*; (G) *CD133*; (H) *NCAM1*; and (I) *PAX6*. (D = day). Significance: * = P < 0.05; ** = P < 0.01; *** = P < 0.001.

**Supplementary Table S1.** Bisulphite sequencing, RT-PCR and quantitative PCR primer sequences and product sizes.

**Supplementary Table S2.** Basal oxygen consumption rates of HSR-GBM1 under demethylation treatment at the undifferentiated or 7 day differentiation towards astrocytes (pmol/per sec/10^6^ cells) ± SEM. Significance: ** = P<0.01; *** = P < 0.001.
